# Pediatric Acute Liver Failure as Presentation of Autoimmune Hepatitis: Learning from a Fatal Case

**DOI:** 10.1097/PG9.0000000000000151

**Published:** 2021-12-10

**Authors:** Lilian H. P. Massabki, Natascha S. Sandy, Adriana M. A. De Tommaso, Maria A. B. Brandão, Gabriel Hessel, Elizete A. Lomazi

**Affiliations:** From the * Pediatric Gastroenterology, Hepatology and Nutrition at University of Campinas, School of Medical Sciences, Campinas, São Paulo, Brazil; † Pediatric Gastroenterology, Hepatology and Nutrition at The Hospital for Sick Children, University of Toronto, Toronto, ON, Canada; ‡ Department of Pediatrics at University of Campinas, School of Medical Sciences, Campinas, São Paulo, Brazil.

**Keywords:** acute liver failure, hepatitis, autoimmune, pediatrics, diagnosis, liver transplantation

## Abstract

Severe acute liver failure (SALF) is a rare condition in children. Up to 50% of the cryptogenic causes of SALF are associated with autoimmune hepatitis (AIH). This report presents a 5-year-old girl with progressive jaundice for 10 days. Her 1999 AIH diagnostic score totaled 11 points, compatible with probable AIH. She fulfilled the SALF criteria and the King’s College criteria for liver transplantation, despite treatment with corticosteroids, and underwent the transplant, but died in the immediate postoperative period due to massive bleeding. Subsequently, the liver-kidney microsome type 1 result was 1:80, increasing the AIH score to 13 points. The final diagnosis was probable AIH type 2, associated with SALF. The biopsy of the explanted liver was compatible with fulminant hepatitis. This report highlights the difficult diagnosis of AIH in SALF, limitations of the diagnostic criteria for SALF in indications for emergency transplantation, and the uncertain therapeutic response produced by corticosteroids.

## INTRODUCTION

Severe acute liver failure (SALF), defined as acute liver damage and the development of encephalopathy within 8 weeks of its onset (1), is rare in children. While idiopathic liver failure represents up to 30% of cases (2), in 2007 Bernal et al, reported that approximately 50% of the cryptogenic causes of SALF are associated with diagnostic criteria (1999 AIH score) for autoimmune hepatitis (AIH) (3,4). Porta et al reported that in hepatology centers in Brazil, in which 828 children with AIH were registered, the probability of developing SALF was 1.6 times greater in AIH type 2 compared with the healthy population (5). The following case is of importance due to the severity of the condition, the importance of AIH as a cause of SALF in children and its difficult diagnosis according to current criteria.

Following exhaustive attempts to contact the family, this case report was approved by the Research Ethics Committee of the State University of Campinas on April 5, 2021, Protocol Number 4,629,727.

## CASE REPORT

**FIGURE 1.**
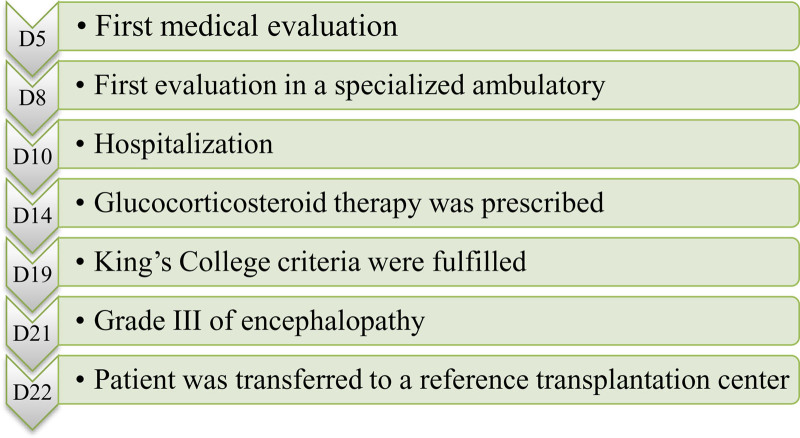
Sequence of clinical events from the first day of jaundice (D0) until the transference of the patient to a reference transplantation center (D22).

A 5 years, 11-month-old girl with no previous comorbidities presented with jaundice for 10 days. Five days later, she was admitted to a local hospital and then referred to a pediatric hepatology clinic with fecal acholia and choluria. Test results are shown in Table 1. Mononucleosis serology was IgM positive and IgG negative, and serum Epstein-Barr virus (EBV) was analyzed by a polymerase chain reaction. The results of a smooth muscle antibody and liver kidney microsome type 1 (anti-LKM-1) tests were not yet known as the sample had been sent to an external laboratory. A liver biopsy was not performed due to changes in the international normalized ratio (INR). After 2 days of consultation, the patient sought emergency care for abdominal pain and distension, in addition to lower limb edema, and was hospitalized. After 4 days, the result of the EBV PCR was negative. The patient’s AIH diagnostic score (1999) was then added (4), including female sex (+2), alkaline phosphatase: alanine aminotransferase ratio (ALP:ALT) < 1.5 (+2), gamma globulin 1.5–2.0 times above the reference value (+2), negative viral markers for acute infection (+3), and no exposure to hepatotoxic drugs (+2), totaling 11 points, which shows probable AIH. Treatment with prednisolone 2 mg/kg/day was started. However, she developed neuropsychomotor agitation in the first week of hospitalization and was diagnosed with grade II hepatic encephalopathy. Computed tomography (CT) of the skull showed no changes. Methylprednisolone 10 mg/kg/day was started to enhance the treatment of probable AIH. The patient presented with progressive INR, increased bilirubin, and decreased transaminases in the second week of hospitalization, with worsening of encephalopathy (grade III) (Table 2). The sequence of clinical events is described in Figure 1. An echocardiogram showed no changes, an abdominal CT scan showed a liver with heterogeneous parenchyma, spleen, and portal vein at the upper limit of normal size, factor V test of 32.4% (RV 70–120%), and negative HIV and Chagas serology. At this time, the patient had already fulfilled the SALF criteria according to the Pediatric Acute Liver Failure Study Group (6). However, due to scarcity of hospital beds in Brazilian public hospitals, transplantation centers usually only admit patients who have already fulfilled the Clichy’s or the King’s College (1) criteria for hepatic transplantation. Therefore, when the child fulfilled the King’s College criteria during the second week of hospitalization, she was transferred to a pediatric liver transplantation center, where she underwent the living-related transplantation (mother donor). However, she died in the immediate postoperative period due to severe bleeding. Biopsy of the explanted liver showed massive lobular necrosis with a severe ductular reaction and necrosis predominantly in the centrilobular region, with relatively mild inflammation, marked cholestasis, and regenerative nodules. The gallbladder presented with mild cholesterolosis, which corresponded to deposits of cholesterol esters and lipids in lamina propria macrophages. Autoantibody results available approximately 40 days after transplantation showed an anti-LKM-1 1:80, increasing the diagnostic score to 13 points. The smooth muscle antibody was 1:20. Thus, the final diagnosis according to the current diagnostic score of 1999 was probable AIH type 2 associated with SALF.

**TABLE 1.** Blood tests from the first evaluation in a pediatric hepatology center

Blood test	Test result (NR)
Gamma glutamyl transferase	150 U/L (5–43 U/L)
Serum albumin	3.8 g/dL (3.2–4.5 g/dL)
Gammaglobulin	2.49 g/dL (0.5–1.4 g/dL)
Antinuclear factor	Negative
Complement system	
C3	0.32 g/L (0.83–1.77 g/L)
C4	0.03 g/L (0.16–0.47 g/L)
Alpha1-antitrypsin	2.07 g/L (1.0–2.0 g/L)
Ceruloplasmin	31 mg/dL (25–63 mg/dL)
Alpha-fetoprotein	46.42 ng/mL (<7.0 ng/mL)
Serologies	
Hepatitis A, B e C	Negatives
Cytomegalovirus	IgM negative e IgG positive
Toxoplasmosis	IgM negative e IgG negative

IgM = immunoglobulin M; IgG = immunoglobulin G; NR = normal range.

**TABLE 2.** Evolution of blood tests from the first day of jaundice (D0) until the transference of the patient to a reference transplantation center (D22)

Day of clinical evolution	AST (U/L)	ALT (U/L)	Total bilirubin (mg/dL)	INR
D5	3054	1553		
D8	2813	1460	13.04	2.24
D10	2137	1168	15.39	2.29
D14	1327	733	16.35	
D19	554	384	18.15	3.21
D21	300	264	23.8	3.27
D22	264	244	22.28	3.15

AST = aspartate aminotransferase; ALT = alanine aminotransferase; INR = international normalized ratio.

## DISCUSSION

AIH is an important cause of liver disease in pediatric patients. In Brazil, it is estimated to correspond to 5–10% of liver diseases in children (5). As for the diagnosis, a score was published in 1999 which might be used to identify AIH. This scoring system is based on the following criteria (4): sex; ALP:AST (or ALT) ratio; serum globulins or IgG above normal; antinuclear antibodies (ANA), smooth muscle antibodies or type 1 liver-kidney microsomal antibodies (anti-LKM-1); antimitochondrial antibodies; hepatitis viral markers; drug history; average alcohol intake; liver histology; autoimmune disease(s); HLA DR3 or DR4; seropositivity for other defined autoantibodies; and response to therapy. If the patient is not under treatment, a score > 15 indicates AIH, while a score of 10–15 points indicates probable AIH. If the patient is already under treatment, a score > 17 may indicate AIH, while a score of 12–17 points indicates probable AIH. In 2018, the European Society for Paediatric Gastroenterology, Hepatology and Nutrition (ESPGHAN) published a position statement that included normal cholangiogram as favorable criteria for AIH diagnosis (7).

It is, however, important to highlight the limitations of this score in fulminant presentations. First, autoantibody results may take a long time to be obtained, delaying treatment, and contributing to a worse prognosis. Liver biopsy to obtain histology is often not performed in SALF, either due to clinical instability or severe coagulopathy. Furthermore, histology can be difficult to interpret in SALF cases, since the fragment may not be representative and present a sampling error. It may even show massive hepatic necrosis, rather than identifying typical AIH patterns (8), as in our case. Since autoimmune disease can account for as much as one-third of all SALF cases (9), it should be considered in the differential diagnosis even in the absence of supportive histologic changes. In this context, considering that histology can increase a diagnostic score by up to 5 points, it is suggested that histological criteria not be considered in cases of SALF and that the score indicating probable or defined AIH be reduced.

The role of corticosteroids as a prognostic modifier in cases of fulminant AIH is controversial since the available studies are limited, with small and poorly characterized cohorts, and varying inclusion criteria (10). Mendizabal et al (11) evaluated 40 adults with fulminant AIH; 17 were treated with corticosteroids, but only seven survived without transplantation. The benefits of high dose corticosteroids to reduce the need for transplantation are still unknown (12), and no studies have been reported for children.

It is notable that our patient did not meet the King’s College criteria (1) in the first week of hospitalization for urgency, delaying her priority for transplant. In addition, she developed grade III encephalopathy only in the second week of hospitalization, during which she still did not meet the Clichy criteria (1). Unfortunately, disease progression was sudden, which likely also contributed to a worse posttransplant outcome.

As for prognosis, a Brazilian study from 2016 compared the outcomes of liver transplantation with living or cadaver donors in children with SALF, reporting a higher incidence of primary organ dysfunction and mortality in cadaver donor transplants (13). However, the results are worse in SALF compared with chronic liver disease in the presence of grade III or IV encephalopathy and with low transaminase levels (10). All these factors were present in our case.

This case report with a fatal outcome underscores the need to reconsider the diagnostic criteria of AIH in SALF. Our case also emphasizes the importance of not waiting to fulfill the Clichy or King’s College criteria for referral to a transplant center, as clinical deterioration may be sudden. It is, furthermore, not prudent to wait for a response to corticosteroids since the dose, route of administration, and indication of use are not well defined in the literature.

## ACKNOWLEDGMENTS

We would like to thank Editage (www.editage.com) for English language editing.
